# STABILISE; treatment of aortic dissection, a single Centre experience

**DOI:** 10.1186/s42155-022-00286-2

**Published:** 2022-01-27

**Authors:** Goran Mitreski, Damian Flanders, Julian Maingard, Domenic Robinson, Jason Chuen, George Matalanis, Siven Seevanayagam, Hong Kuan Kok, Dinesh Ranatunga, Hamed Asadi, Duncan Mark Brooks

**Affiliations:** 1 Department of Radiology, Austin Health, 145 Studley Rd, Heidelberg, VIC 3084 Australia; 2 Department of Radiology, Monash Health, Clayton, Victoria Australia; 3 Department of Vascular surgery, Austin Health, Heidelberg, Victoria Australia; 4 Department of Cardiac surgery, Austin Health, Heidelberg, Victoria Australia; 5 Department of Radiology, Northern Health, Epping, Victoria Australia; 6 Department of Radiology, Austin Health, Heidelberg, Victoria Australia

## Abstract

**Purpose:**

To outline the process of the STABILISE technique and its use; reporting patient outcomes and midterm follow up for complicated aortic dissection.

**Materials and methods:**

Single centre retrospective analysis from January 2011 to January 2021 using the STABILISE technique which utilises balloon assistance to facilitate intimal disruption and promote aortic relamination.

**Results:**

Sixteen patients underwent endovascular aortic repair with the STABILISE technique for aortic dissection over the study period. Fourteen patients (14/16; 88%) had acute dissection. Two of 16 (12%) were chronic. The median age of the patient cohort was 61 years (range 32–80 years) and consisted of a male majority (*n* = 11; 69%). The median time from diagnosis to intervention was 5 days (1–115 days; IQR 1–17.3). More than half (56%) had surgical repair of a acute type A aortic dissection prior to radiological intervention. The procedure was technically successful with no procedural mortality. Two patients were lost to follow up and two died in the post-operative period. Twelve patients had ongoing follow up with an average number of 2.9 ± 1.6 scans performed. Follow up was available in thirteen patients (81%) with a median follow up period of 1097 days (IQR 707–1657). The rate of re-intervention (*n* = 2/16; 13%) requiring additional stenting was in line with published re-intervention data (15%). Follow up showed a reduction in false lumen size following treatment with total luminal dimensions remaining stable over the follow-up period.

**Conclusion:**

The STABILISE technique as a procedure for complicated aortic dissection, either acute or chronic, appears safe with stable mid-term aortic remodelling and patient outcomes.

**Level of evidence:**

Level 3, Retrospective cohort study.

## Introduction

Aortic dissection is an important cause of morbidity and mortality worldwide. Acute aortic dissection, occurring within a 2 week period of symptom onset (Nienaber & Powell, 2012) is part of the spectrum of acute aortic syndrome. This also includes intramural haematoma, penetrating aortic ulcer and symptomatic or ruptured aortic aneurysm. (Erbel et al., 2014) The two common classifications for aortic dissection include the DeBakey system (types I, II and III) (Debakey et al., 1965) and the Stanford systems (types A and B) (Daily et al., 1970), these are classified based on involvement of the ascending aorta (DeBakey I-II, Stanford A) or sparing the ascending aorta and arch vessels (DeBakey III, Stanford B).

First described in 1994 by Dake et al., (Dake et al., 1994) thoracic endovascular aortic repair (TEVAR) and abdominal endovascular aortic repair (EVAR) have been widely used in the management of acute aortic syndromes. Endovascular repair provides benefit as it avoids major surgical incisions, aortic cross-clamping, reduces procedural time, decreases blood loss, and decreases end-organ ischaemia. (Dangas et al., 2012; Walsh et al., 2008) Reduced post-operative mortality (30 day; 7.9% vs 20% and 1 year; 8.7% vs 17%), in addition to reduced procedural complications, have also been demonstrated in those undergoing TEVAR compared with open repair (Harky et al., 2020; Hsieh et al., 2019). Despite these benefits, meta-analyses have shown TEVAR to be associated with pooled reintervention rates of 15%; reasons including, endoleak (33.2%), false-lumen perfusion and aortic dilation (19.8%), and new dissection (6.9%). (Faure et al., 2014; Zhang et al., 2016).

In an attempt to promote aortic remodelling and eliminate false lumen perfusion, Hofferberth et al. (Hofferberth et al., 2014) introduced “stent assisted balloon-induced intimal disruption and relamination in aortic dissection repair” (STABILISE). The approach aims to produce a single aortic channel in the thoracic/distal aorta using a compliant balloon to extend the fenestration along the length of the dissected segment and reoppose the layers of the dissected aortic wall using self-expanding stents. Two similar approaches performed prior to STABILISE were the PETTICOAT (Provisional Extension to Induce Complete Attachment) (Mossop et al., 2005; Nienaber et al., 2006; Rong et al., 2019) and STABLE (Staged Total Aortic and Branch Vessel Endovascular) (Hofferberth et al., 2012b) techniques. The PETTICOAT technique provided internal support to the intima from within the true lumen, reducing intimal flap motion. This lowered the risk of new tears at the distal end of the covered stent, and reduced movement of blood within the false lumen promoting stasis and thrombosis. This approach was effective in reducing the risk of aneurysmal dilatation of the thoracic false lumen. The STABLE technique established further reduction in false lumen flow by occluding remaining small fenestrations using covered stents, coils, and vascular plugs. It had been shown to be effective in controlling abdominal as well as thoracic false lumen growth but was technically difficult and typically required multiple procedures over an extended time period. Both used proximal covered and distal bare stents but did not include balloon fenestration. The STABLE technique included the use of branch vessel covered stents and other techniques for closing any remaining communications between the true and false lumens. More recently, the STABLE II technique has been published demonstrating improved outcomes post treatment of acute complicated type B aortic dissection. (Lombardi et al., 2020)

As a novel technique, publications and case numbers utilising the STABILISE technique remain small in volume. A retrospective review by Faure et al. in 2018 (Faure et al., 2018) sought to validate the initial findings by the Hofferberth group assessing 41 patients. Their results supported the STABILISE approach as a safe and reproducible technique with encouraging midterm results.

In this study, we report the outcomes of a consecutive series of patients undergoing the STABILISE technique for complicated aortic dissection at a quaternary teaching hospital in Australia.

## Methods

A single-centre retrospective review was undertaken for all patients who underwent endovascular management for aortic dissection using the STABILISE technique (intimal balloon fenestration and attempt aortic relamination as described above). Ethics approval was granted by the local institutional ethics and review board; reference number HREC/53590. STROBE cohort reporting guidelines have been utilised. (von Elm et al., 2008)

### Patients

Between January 2011 to January 2020, all patients who underwent endovascular repair (EVAR and TEVAR) for type A and B dissection using the STABILISE technique were included in the study. Indications for intervention included aortic rupture, visceral organ malperfusion, progressive false lumen growth of more than 5 mm over serial computed tomographic (CT) scans, total aortic dimension of more than 40 mm, refractory hypertension and persistent pain. STABILISE was utilised in patients if there was persistent false filling after initial deployment of a covered aortic stent. Patient details including demographics, dissection morphology, prior interventional history, operation details and post-operative follow-up were recorded.

### Endovascular procedure and prosthesis

The Zenith Dissection Endovascular System (Cook Medical Inc., Bloomington, Ind) is a modular system specifically designed to treat aortic dissection, consisting of a proximal component, the Zenith TX2 TAA Endovascular Graft, and a distal component, the Zenith Dissection Endovascular Stent. A detailed description of the Zenith Dissection Endovascular System has been previously reported. (Hofferberth et al., 2012a; Melissano et al., 2008; Mossop et al., 2005).

### Technique

The procedure is performed under general anaesthesia in an Angiography suite or hybrid theatre with DSA imaging. A 6 French sheath is placed in the left common femoral artery (CFA) and a 5 Fr 100 cm measuring pigtail catheter advanced over a guidewire into the aortic true lumen proximal to the dissection, commonly into the ascending aorta. Right common femoral artery access is gained either surgically or percutaneously using a preclose technique with Perclose ProGlide system™ (Abbott). Using an 8Fr arterial sheath, an angled catheter and Terumo glidewire® (Terumo) are advanced through the true lumen to the ascending aorta and the wire exchanged for a 300 cm double curve Lunderquist® extra-stiff wire (Cook). The Zenith TX2 stent graft (covered stent) is introduced percutaneously over the Lunderquist wire and deployed in a landing zone more proximal to the proximal extent of the dissection. Zenith Dissection (uncovered) Stents (Cook) are then deployed with approximately 20 mm overlap throughout the dissected aortic segment. Care is taken to avoid stent overlap in the visceral segment of the abdominal aorta. Balloon dilatation (Coda® balloon; Cook) commences within the TX2 (covered) stent graft using the 46 mm Coda® balloon and is continued sequentially in an overlapping pattern through the stent graft and bare stent, changing to the 32 mm balloon as required depending on distal aortic diameter. When dissection continues into the common iliac arteries, these are treated using large self-expanding nitinol stents (Zilver® Vena; Cook). Balloon dilatation within iliac arteries is achieved either with an angioplasty balloon sized to the total iliac diameter or gentle partial inflation of the 32 mm Coda® balloon. Balloon dilatation is achieved via a pressure inflator titrating balloon expansion whilst simultaneously screening via fluoroscopy. The authors found the balloon setup described above to work consistently with most patients, changing balloon diameters distally within the aorta/iliacs according to arterial dimensions unique to each patient.

Depending on aortic angiogram appearances, the visceral arteries were stented with balloon expandable stents either covered or uncovered to treat tear extension into these branches.

### Aortic remodelling

**Fig. 1:**
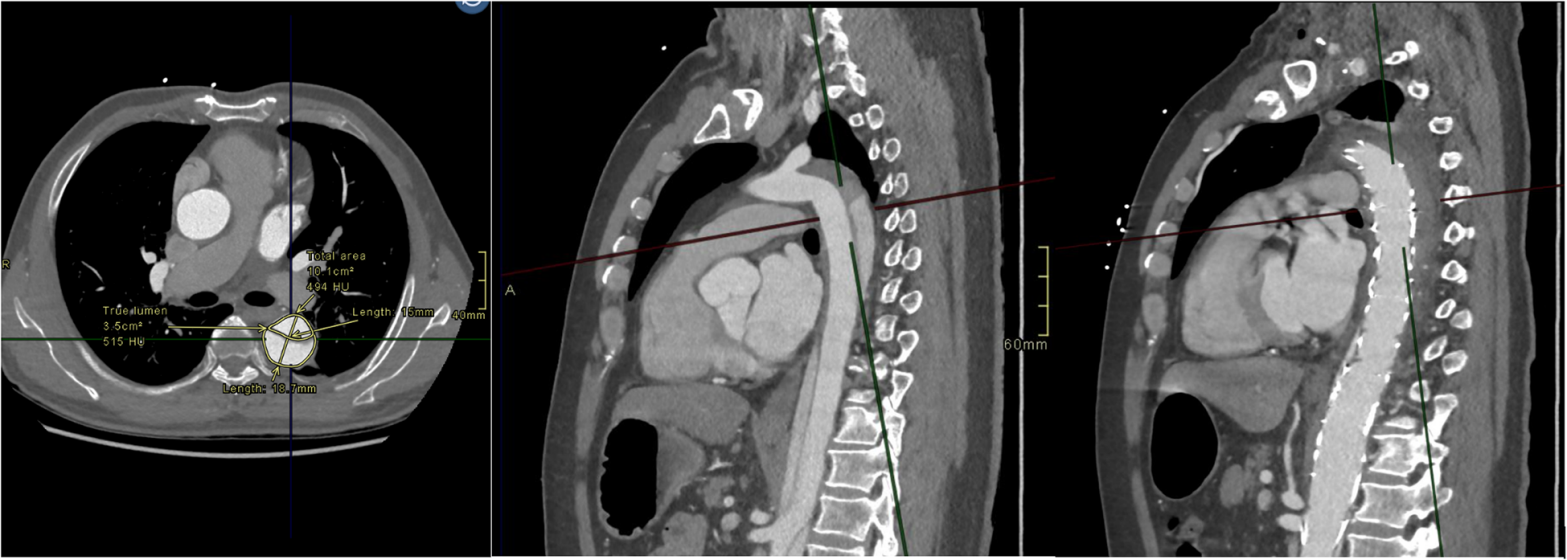
MPR at the level of the carina with selected fluoroscopic images showing obliteration of the false lumen at the level of the arch/descending aorta following covered stent deployment

**Fig. 2:**
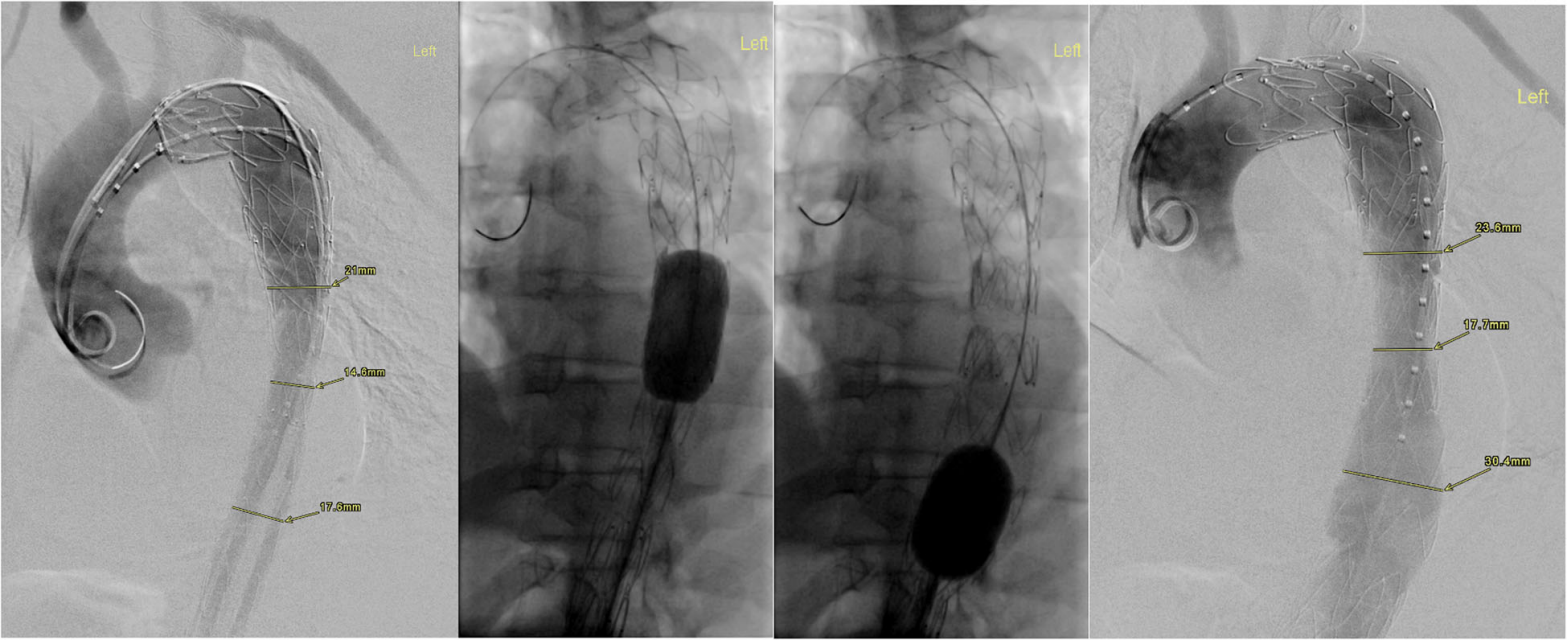
Selected fluoroscopic images of the aortic arch and descending aorta, pre and post balloon fenestration demonstrate true lumen narrowing with appropriate patency post balloon dilatation

**Fig. 3:**
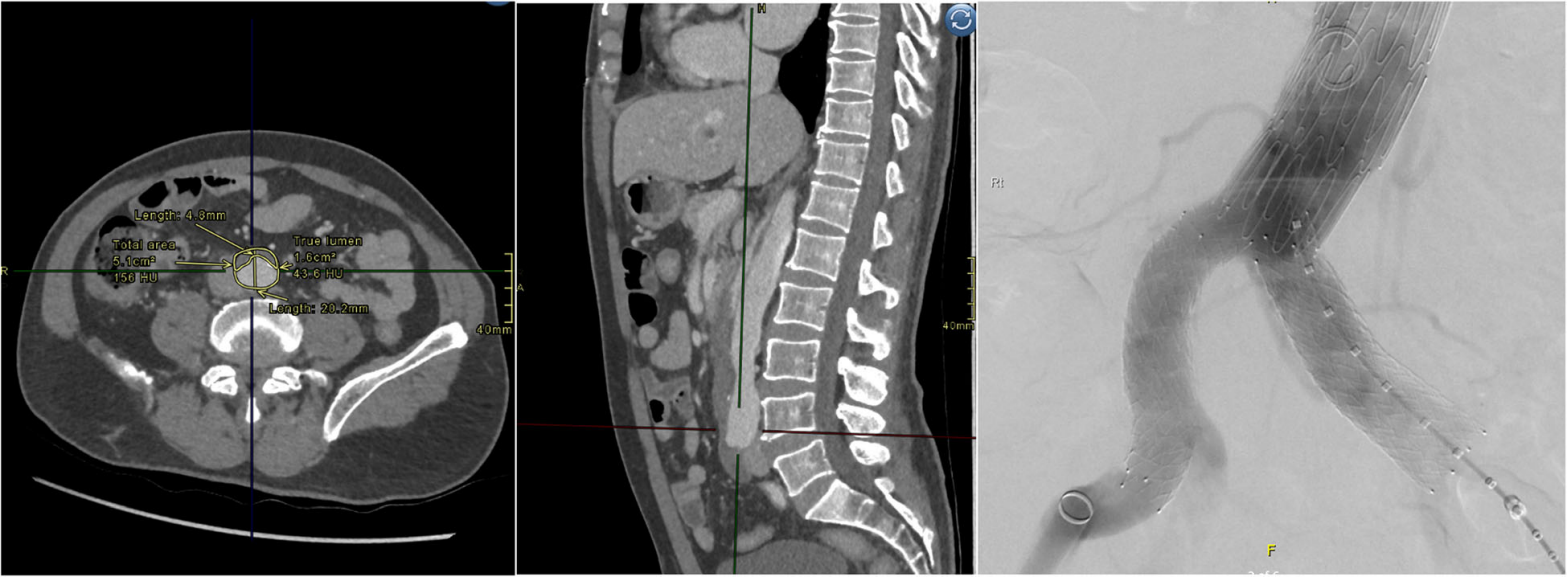
MPR at the level of the aortic bifurcation. Bilateral iliac stents have been deployed to correct iliac dissection, captured during stent deployment

Aortic remodelling post-STABILSE was evaluated using the index pre-preprocedural and subsequent follow-up imaging studies. Aortic cross-sectional diameters and luminal values were measured, including true lumen, false lumen, and total luminal transverse dimensions on standard axial acquisitions. The aortic level measurements were standardised to include the level of the carinal bifurcation, coeliac artery origin, renal artery origin at the midpoint of the left and right renal ostium and at the aortic bifurcation (iliac vessel origins). Cross-sectional area orthogonal to the centreline of the vessel was obtained using post-processing software suite (AGFA IMPAX® client) (*see* Figs. 1, 2 and 3).

### Data analysis

Data was assessed for normality using the Shapiro-Wilk normality test and expressed as numbers (%) for categoric values and median (interquartile [IQR] range) or mean (± standard deviation) for continuous variables. As the data was not normally distributed (i.e., non-parametric), Mann-Whitney U testing was utilised to analyse the continuous variables.

## Results

Sixteen patients underwent endovascular aortic repair with the STABILISE technique for aortic dissection over the study period (2011–2020). The median age of the patient cohort was 61 years (range 32–80 years) and consisted of a male majority (*n* = 11; 69%). The median time from diagnosis to intervention was 5 days (1–115 days; IQR 1–17.3). A breakdown of the Type A and B cases are demonstrated below in Table 1.

**Table 1:** Dissection characteristic of patients who underwent STABILISE intervention

Sex/Age (y)	Dissection type	Days post diagnosis (d)	Indications for stabilise
F/68	Post type A repair: Acute	14	False lumen growth and unilateral renal malperfusion
M/58	Post type A repair: Acute	2	Mesenteric malperfusion
M/75	Post type A repair: Acute	0	Unilateral renal and aortoiliac malperfusion
M/66	Post type A repair: Acute	13	False lumen growth, rupture, enlarging aortic dimensions and unilateral renal malperfusion
M/48	Post type A repair: Acute	13	False lumen growth, unilateral renal and aortoiliac malperfusion
M/42	Post type A repair: Acute	1	Unilateral renal and aortoiliac malperfusion
F/51	Post type A repair: Acute	7	False lumen growth
M/64	Post type A repair: Acute	4	Rupture
F/80	Post type A repair: Acute	1	Unilateral renal malperfusion
M/58	Acute type B	3	Refractory hypertension, pain, bilateral renal and aortoiliac malperfusion.
F/32	Acute type B	2	Enlarging aortic dimensions, refractory pain and hypertension
M/67	Acute type B	0	Rupture and enlarging aortic dimensions
M/81	Acute type B	27	Enlarging aortic dimensions, refractory hypertension and unilateral renal/aortoiliac malperfusion.
M/69	Acute type B	1	Bilateral renal malperfusion
F/55	Post type A repair: Chronic	115	Enlarging aortic dimensions
M/52	Post type B repair: Chronic	90	Enlarging aortic dimensions

Nine of sixteen patients (56%) had surgical repair of the acute type A aortic dissection prior to radiological intervention. One of the acute type A presentations had a previous Bentall’s procedure for congenital bicuspid valve (*n* = 1; 6%). One patient with a previous history of type A repair had a diagnosis of Marfan’s syndrome, presenting acutely with a Type B dissection One patient had prior sternotomy for coronary artery bypass grafting (CABG). Table 2 outlines the patient demographics and details of the dissection morphology, vessels involved, and vessels stented.

**Table 2:** Demographics of patients who underwent STABILISE intervention

Demographics			Total n (%)
Median age, years (IQR)			61 (52–69)
Male			11 (69)
Ethnicity	Caucasian		15 (94)
	Asian		1 (6)
Patient history	Connective tissue disease		1 (6)
	Hypertension		8 (50)
	Bicuspid aortic valve		1 (6)
	Smoker		4 (25)
	Previous cardiac surgery	Type A dissection	1 (6)
		Aortic valve + arch	1 (6)
		CABG	1 (6)
Dissection morphology	Post-acute type A repair		9 (56)
	Delayed post type A repair		1 (6)
	Acute type B		5 (31)
	Chronic type B		1 (6)
Indications for endovascular repair	Visceral malperfusion		11 (75)
	Aortofemoral malperfusion		8 (50)
	Rupture		3 (19)
	False lumen growth > 5 mm		4 (25)
	Max aortic size > 40 mm		5 (31)
	Refractory pain		3 (19)
	Refractory hypertension		3 (19)
Vessel involvement	Coeliac		1 (6)
	SMA		3 (19)
	Left renal		11 (69)
	Right renal		3 (19)
	Infrarenal		16 (100)
	Iliacs		12 (75)
Involved vessels stented	Coeliac		0
	SMA		1/3 (33)
	Left renal		5/11 (45)
	Right renal		2/3 (67)
	Infrarenal		15/16 (94)
	Iliacs		8 /12 (67)

*IQR,* interquartile range; *CABG,* coronary artery bypass grafting; *SMA,* superior mesenteric artery

### Clinical follow up

Pre-procedural imaging was available in 14 of 16 patients (87%). Two patients died within 30 days of the procedure (day 1 and day 27). One patient (acute type A) died due to multiple associated medical problems including pulmonary haemorrhage, end-organ ischaemia and marked coagulopathy whilst the second patient (acute type B) died from complications of renal ischaemia and failure, which preceded the STABILISE intervention. More recent larger sample studies assessing treatment of acute type B dissection have demonstrated 30-day mortality at 6.8%. (Lombardi et al., 2020)

Two patients were lost to follow up, one at 87 days, the other at 3.5 years post procedure.

The average number of scans performed for patient follow up was 2.9 ± 1.6 scans. Follow up was available in thirteen patients (81%) with a median follow up period of 1097 days (IQR 707–1657).

No patient deaths were recorded from 30 days post procedure until the end of the review period (January 2020). One patient (8%) required further operative intervention to manage delayed endo-leak at 5.8 years post initial procedure. The remaining 12 patients were free from procedure-related complications including stent rupture, stent migration, vessel occlusion or re-dissection.

### Aortic remodelling

**Fig. 4:**
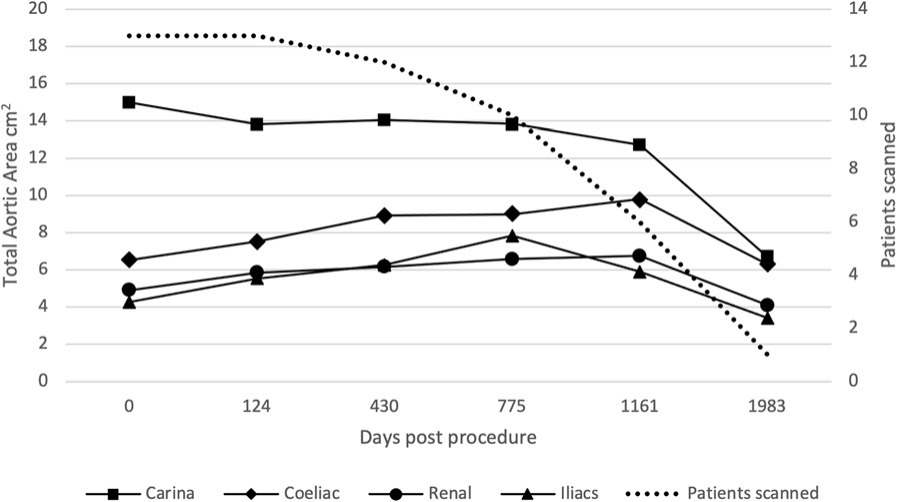
Average total aortic area (cm^2^) excluding cases requiring reintervention

**Fig. 5:**
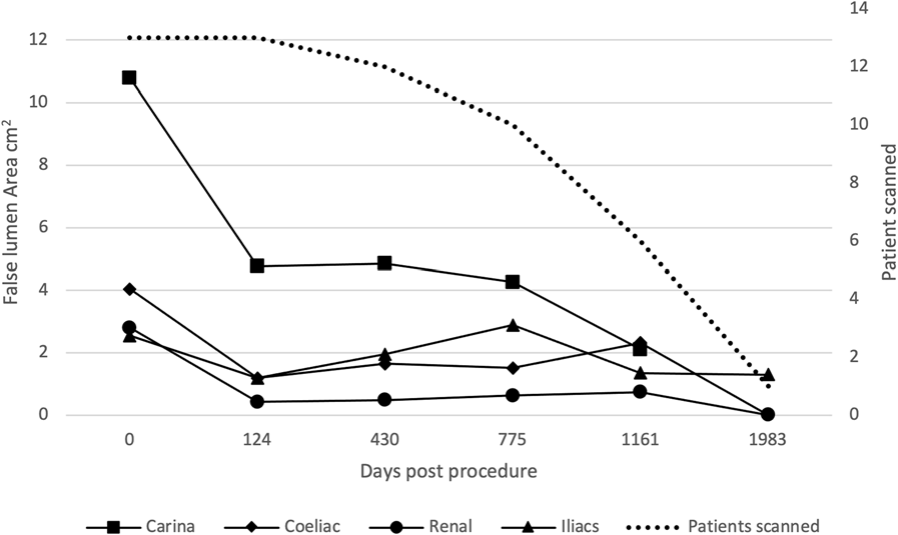
Average false lumen aortic area (cm^2^) excluding cases requiring reintervention

**Fig. 6:**
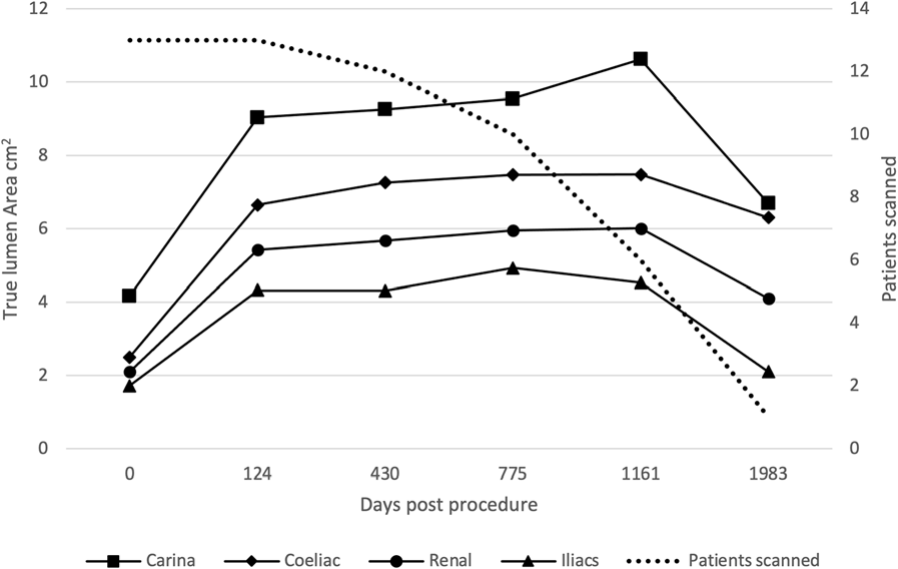
False lumen aortic area (cm^2^) excluding cases requiring reintervention

**Fig. 7:**
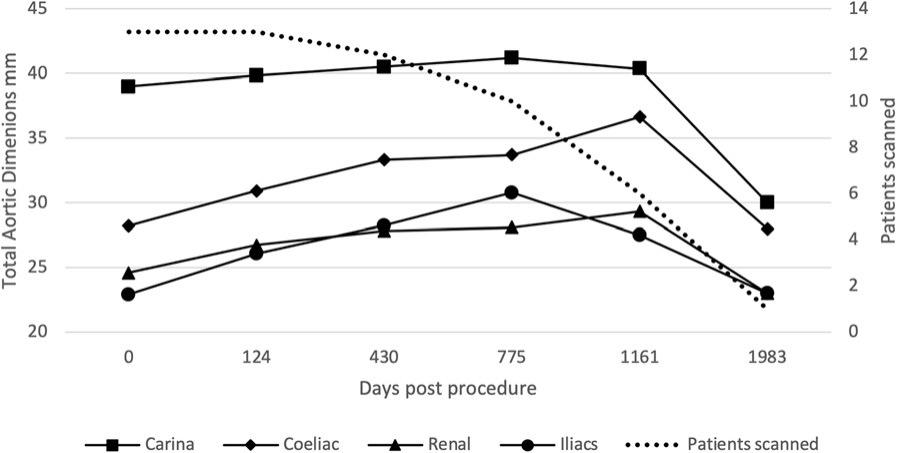
Total aortic dimensions (mm) excluding cases requiring reintervention

Figures 4, 5, 6 and 7 outline the aortic area measurements, including diameter and area in the pre-and post-intervention period in those treated successfully. In line with previously reported studies, the maximum aortic dimensions were observed in the thoracic aorta at the level of the carina, with pre interventional mean aortic areas measuring 15 ± 15 cm^2^.

**Fig. 8:**
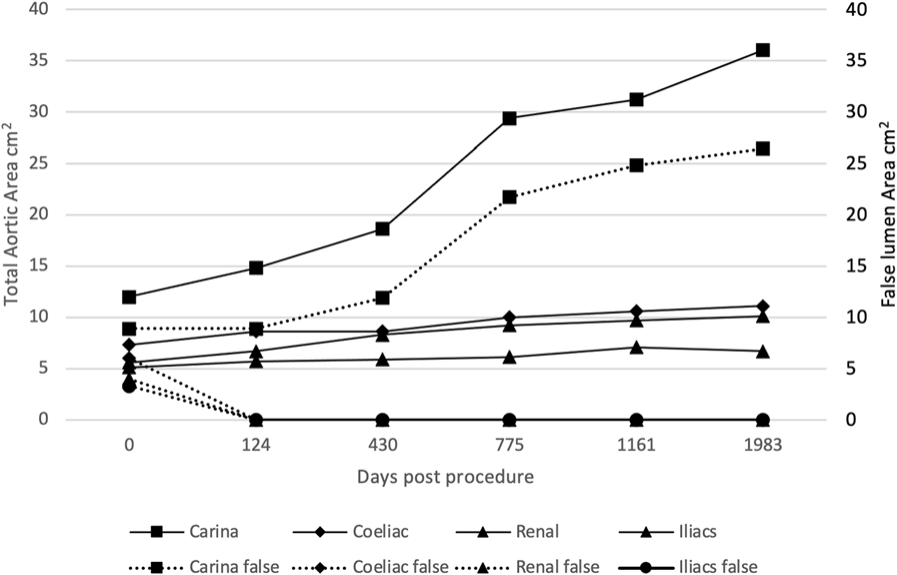
Total and false luminal aortic area (cm^2^) in one patient with progressive endoleak

A late increase in diameter at the carinal level was noted in a single case with persistent endoleak (Fig. 8). The other 12 cases with long term follow up all showed a reduction in false lumen size following treatment. Total luminal dimensions remained stable over the follow-up period, allowing for an increased carinal level area measurement secondary to the endo-leak described above, which required re-intervention. True lumen area and total aortic dimensions, accounting for the increased false luminal diameter in the re-operated patient were otherwise stable with no significant dilatation; *p* = 0.83 [> 0.05]; (Kruskal-Wallis nonparametric testing).

### Aortic reintervention

Two out of the sixteen patients (13%) required further intervention to manage false lumen enlargement. Both patients had filling of the false lumen at the time of follow up scanning. One patient was retreated 14 days after the initial procedure with deployment of an additional stent graft. The other patient with the delayed endoleak (Fig. 8) had two further treatments, at 286 and 2066 days post procedure. The first treatment was deployment of an Amplatzer™ Vascular Plug II (Abbott Vascular) occluder device in the left subclavian artery to prevent retrograde filling of the false lumen. Subsequently, a stent graft was deployed to completely obliterate the false lumen in the thoracic aorta. No new sites of dissection were identified in the interventional group through follow up imaging. The graphs detailed above demonstrate the evolving false lumen in the patient described above.

## Discussion

The high technical success rate and safety profile of this STABILISE cohort is in keeping with previous results. (Hofferberth et al., 2012a) A review of the current literature outlined below (see Tables 3 and 4) summarises the use of STABILISE technique in complicated aortic dissection.

**Table 3:** Updated studies assessing the STABILISE technique

Study	Year	Journal	Study Design	Data Collection	Centre
**Soler et al (Soler et al.,** 2021**)**	2021	European Journal of Vascular Endovascular Surgery	Observational cross-sectional	Retrospective	Single
**Zhong et al (Zhang et al.,** 2016**)**	2021	Journal of Cardiovascular Interventional Radiology	Observational cohort	Retrospective	Single
**Faure et al (Hofferberth et al.,** 2012a**)**	2020	Journal of Cardiovascular surgery	Observational cohort	Prospective	Single
**Lopes et al (Nienaber et al.,** 2006**)**	2019	European Journal of Vascular and Endovascular surgery	Case series	Retrospective	Single
**Faure et al (Harky et al.,** 2020**)**	2019	Journal of Thoracic and Cardiovascular surgery	Observational cross-sectional	Retrospective	Single
**Kahlberg et al (Mossop et al.,** 2005**)**	2019	Journal of Cardiovascular surgery	Observational cohort	Prospective	Single
**Faure et al (Faure et al.,** 2020**)**	2018	European Journal of Vascular and Endovascular surgery	Observational cross-sectional	Retrospective	Single
**Faure et al (Faure et al.,** 2018**)**	2018	Journal of Thoracic and Cardiovascular surgery	Observational cross-sectional	Retrospective	Single
**Melissano et al (Rong et al.,** 2019**)**	2018	Journal of Vascular surgery	Observational cohort	Prospective	Single
**Hofferberth et al (Lombardi et al.,** 2020**)**	2014	Journal of Thoracic and Cardiovascular surgery	Observational cross-sectional	Retrospective	Single

**Table 4:** Demographics and treatment characteristics of the reviewed articles

Study	Year	Patients (n)	Median age (y)	Age range (y)	Sex (%M)	Organ ischaemia (n)	Aortic dimensions > 40 mm (n)	Hypertension (n)	Iliac extension (n)	Post-operative mortality (30 days)	Mean follow up period (months)	Re-intervention > 30 days
**Soler et al (Soler et al.,** 2021**)**	2021	8	49	29–64	x	2	x	x	x	0	15	2
**Zhong et al (Zhang et al.,** 2016**)**	2021	11	54	31–82	76	x	x	x	x	1	31	1
**Faure et al (Hofferberth et al.,** 2012a**)**	2020	17	61	46–66	88	x	17	x	15	0	17	2
**Lopes et al (Nienaber et al.,** 2006**)**	2019	2	69.5	69–70	100	0	2	2	x	0	x	0
**Faure et al(Harky et al.,** 2020**)**	2019	16	56	43–65	75	13	3	x	16	1	8	1
**Kahlberg et al(Mossop et al.,** 2005**)**	2019	14	61.2	53–69	93	9	3	5	3	0	12.3	0
**Faure et al (Faure et al.,** 2020**)**	2018	41	50	23–87	83	20	10	2	x	1	12	8
**Faure et al(Faure et al.,** 2018**)**	2018	7	47	23–70	86	2	5	x	6	0	15	1
**Melissano et al (Rong et al.,** 2019**)**	2018	10	62.6	55–70	100	6	1	3	2	0	7.2	0
**Hofferberth et al (Lombardi et al.,** 2020**)**	2014	11	50	35–67	91	5	1	x	x	1	19	0

In our series, medium term survival was excellent. Aortic dimensions remained stable through the study period (up to 5 years). Re-intervention was required in 2 patients for persistent endoleak. Failure to successfully control the endoleak in one of the patients was associated with ongoing growth of the false lumen and total aortic diameter. This reinforces the role of complete false lumen exclusion in successful management of this condition. Ongoing clinical and radiological follow up remains important to detect and manage any persistent growing false lumen. Further, there was no commonality in endoleak location between the two patients, as one was seen along the most inferior margin of the aortic stent, the other at the thoracic junction. Our rate of re-intervention was similar to meta-analyses described above suggesting 15% re-treatment. (Zhang et al., 2016) No long term sequalae have been identified in the two patients who required reintervention. When performed with open surgical repair for acute type A dissection, the STABILISE technique did not add morbidity. Although our study has shown a 12.5%, 30 day mortality rate, no direct deaths were attributed to the STABILISE procedure. Both deaths were secondary to pre-existing ischaemia and organ dysfunction. It is difficult to infer meaningful comparison to more recent studies demonstrating a 6.8% mortality post STABILISE (Lombardi et al., 2020) given small sample size of complicated type B dissection in our study cohort (*n* = 5). No peri-procedural complications including groin haematoma or iatrogenic arterial dissection were identified.

Although most of the patients were treated in the acute phase, two chronic dissections were treated successfully at 90 days and 115 days. Other reports suggest that treatment beyond this time may be associated with difficulty in disrupting the intimal flap although still safe and efficacious. () The authors consider the hyperacute phase (within 24–48 h) post dissection to carry too high of a risk of aortic rupture whilst delayed treatment in the chronic phase, considered after 12 weeks, reduces intimal pliability and possible successful relamination. In any case, careful balloon remodelling with high dose fluoroscopy and wire control is paramount in ensuring appropriate fenestration. Another reason for avoiding late treatment is the difficulty in managing aortic dilation due to the inability to obtain an appropriate stent graft apposition site once the distal thoracic aorta diameter approaches 46 mm. Despite risks of aortic rupture, there is limited data published substantiating this risk with only one rupture identified in the literature during aortic remodelling. (Zhong et al., 2021) STABILISE has also been utilised in cases of connective tissue disease (Soler et al., 2021) with authors suggesting close follow up due to aneurysmal evolution at the bare stent level.

The aim of STABILISE technique goes beyond the PETTICOAT technique with the goal of complete false lumen obliteration. Although STABLE also aims to completely obliterate false lumen flow, STABILISE is a simpler more stereotyped technique which is more easily applied to a range of patients and operator experience. Using a compliant balloon to extend fenestration along the whole length of the dissected segment and bare stents to reappose the intimal flap to the outer media restores physiological pressure and flow to a single aortic lumen. This appears to remove the pathophysiological processes which drive progressive false lumen dilatation in the abdominal aorta as well as the thoracic false lumens.

Although the results are favourable and on par with other centres from around the world, we acknowledge the limitations of a small sample size and single centre retrospective experience. Further single and multi-centre studies would be useful.

## Conclusion

The STABILISE technique as a procedure for complicated aortic dissection either acute or chronic appears safe with stable mid-term aortic remodelling and patient outcomes. The rate of re-intervention in our series is in line with published data. The STABILISE procedure can be considered where there is extensive dissection necessitating reconstitution of inline aortic flow. Further multi-centre prospective studies would be of use to validate the indications and long-term outcome.

## Data Availability

Yes

## Abbreviations

TEVAR: Thoracic endovascular aortic repair
EVAR: Endovascular aortic repair
STABILISE: Stent assisted balloon induced intimal disruption and relamination in aortic dissection repair
PETTICOAT: Provisional Extension to Induce Complete Attachment
STABLE: Staged Total Aortic and Branch Vessel Endovascular
CABG: Coronary artery bypass grafting
IQR: Interquartile range
